# Effect of Ulinastatin Combined With Dexmedetomidine on Postoperative Cognitive Dysfunction in Patients Who Underwent Cardiac Surgery

**DOI:** 10.3389/fneur.2019.01293

**Published:** 2019-12-19

**Authors:** Meiyan Zhou, Yi Lyu, Yangzi Zhu, Teng Jiang, Congyou Wu, Jianping Yang, Liwei Wang

**Affiliations:** ^1^Department of Anesthesiology, Intensive Care Medicine and Pain Medicine, First Affiliated Hospital of Soochow University, Suzhou, China; ^2^Department of Anesthesiology, Xuzhou Central Hospital, Xuzhou, China; ^3^Department of Anesthesiology, Minhang Hospital, Fudan University, Shanghai, China

**Keywords:** ulinastatin, dexmedetomidine, cardiac surgery, inflammatory response, postoperative cognitive dysfunction

## Abstract

**Background:** Recent studies have shown that early diagnosis and intervention promote the patient's good prognosis. For patients who underwent cardiac surgery and require extracorporeal circulation support, the incidence of postoperative cognitive dysfunction (POCD) is higher than in other types of surgery due to greater changes in brain perfusion compared with normal physiological conditions. Recent studies have confirmed that the use of ulinastatin or dexmedetomidine in the perioperative period effectively reduces the incidence of POCD. In this study, ulinastatin was combined with dexmedetomidine to assess whether the combination of the two drugs could reduce the incidence of POCD.

**Methods:** One hundred and eighty patients with heart valve replacement surgery undergoing cardiopulmonary bypass from August 2017 to December 2018 were enrolled, with age 60–80 years, American Society of Anesthesiologists (ASA) grades I–III, education level above elementary school, and either gender. According to the random number table method, patients were grouped into ulinastatin + dexmedetomidine (U+D) group, ulinastatin (U) group, dexmedetomidine (D) group, and normal saline (N) control group. Group U was pumped 20,000 UI/kg immediately after induction and the first day after surgery, group D continued to pump 0.4 μg/kg/h from induction to 2 h before extubation, group U+D dexmedetomidine 0.4 μg/kg/h + ulinastatin 20,000 UI/kg, and group N equal volume of physiological saline. The patients were enrolled with Mini-Mental State Examination (MMSE) before surgery. The cognitive function was assessed by Montreal Cognitive Assessment (MoCA) on the first day before surgery and on the seventh day after surgery. Inflammatory factors, such as S100β protein, interleukin (IL)-6, matrix metalloproteinase (MMP)-9, and tumor necrosis factor (TNF)-α, were detected in peripheral blood before anesthesia (T0), immediately after surgery (T1), and immediately after extubation (T2).

**Results:** One hundred and fifty-four patients enrolled in this study. Compared with group N, the incidence of POCD in group U+D was the lowest (*P* < 0.05), followed by group U and group D. Group U+D had the lowest concentration of inflammatory factors at the T1 and T2 time points, followed by group U and group D.

**Conclusions:** Both ulinastatin and dexmedetomidine can reduce the perioperative inflammatory response and the incidence of POCD in patients with heart valve surgery, and their combination can better reduce the incidence of POCD.

## Introduction

Postoperative cognitive dysfunction (POCD) is a common central nervous system complication after major surgery in elderly patients (1). According to reports, the incidence of POCD can be as high as 30–60% 1 week after cardiac surgery (2). The occurrence of POCD prolongs the hospitalization time of patients, reduces the quality of life of patients, increases postoperative mortality, and causes a serious burden on individuals and society (3). A large number of studies have shown that (4) inflammatory response plays a key role in the pathophysiological mechanism of POCD, and inhibiting perioperative inflammatory response can significantly reduce the occurrence of POCD and pathological damage of the brain (5, 6).

Ulinastatin is a serine protease inhibitor extracted from human urine, inhibiting the activity of various enzymes to reduce inflammation (7), stabilizing lysosomal membranes and scavenging free radicals. Clinically, the application of ulinastatin can reduce the level of inflammatory factors, thereby reducing the incidence of POCD to some degree (8).

Dexmedetomidine is an α-2 adrenergic receptor agonist that calms, analgeses, and inhibits sympathetic nervous system activity. It can inhibit inflammatory response and stress response, reduce neuronal toxicity and apoptosis, and promote brain protection through synapse formation and neurotrophic nutrition. Multiple studies have shown that perioperative use of dexmedetomidine can reduce the incidence of POCD (9, 10). Previous studies have confirmed that dexmedetomidine and ulinastatin can each inhibit inflammation and reduce the incidence of POCD in patients undergoing cardiac surgery, but whether the combination of the two drugs is more conducive has not been reported. Therefore, this study was to see if the combination of the two drugs can reduce the incidence of POCD in patients with heart valve surgery.

## Materials and Methods

### Patients

This study was a prospective, randomized, double-blind trial, which was registered with the Chinese Clinical Trial Register (Registration number: ChiCTR-IPR-17012544). The study protocol was approved by the Ethics Committee of Xuzhou Central Hospital. Written informed consent was obtained from each patient. We enrolled 180 patients older than 60 years (age ≤80 years) with ASA status of I–III. Thirty community volunteers older than 60 years (age ≤80 years) were examined to exclude the practice effect of repeated neuropsychological testing.

Exclusion criteria were Mini-Mental State Examination (MMSE) score ≤23, acute or chronic infectious diseases, taking anti-inflammatory drugs, or immunosuppressants, a stroke in the prior 6 months or any other central nervous system diseases, peptic ulcer disease, stay in intensive care unit ≥3 days, body mass index (BMI) >35, severe deafness or vision problems, illiteracy and/or communication difficulties related to pronunciation or dialect, postoperative delirium, and refusal or unexpected discharge. We interviewed the patients on the day before surgery and collected baseline data, including age, sex, gender, BMI, past medical history, education history, and MMSE.

The included patients were randomized into a control group (Group C), a dexmedetomidine group (Group D), an ulinastatin group (Group U), and an ulinastatin+dexmedetomidine group (Group U+D). The randomization sequence without stratification was generated by a computer and sealed with consecutively numbered envelopes. The nurses were responsible for preparing ulinastatin, dexmedetomidine, or normal saline. Patients and investigators were all blind to group allocation until the final statistical analysis was completed.

**Figure 1:**
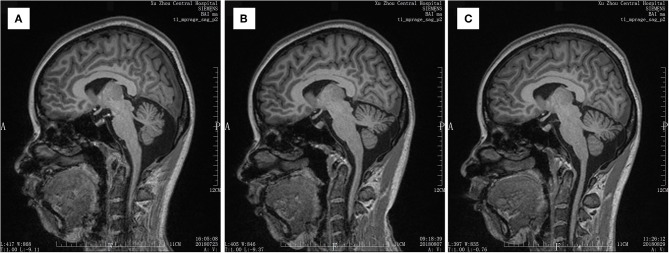
MRI T1 scan result of a patient. **(A)** Scan result before the surgery. **(B)** Scan result at 7 days after the surgery. **(C)** Scan result at 30 days after the surgery.

**Figure 2:**
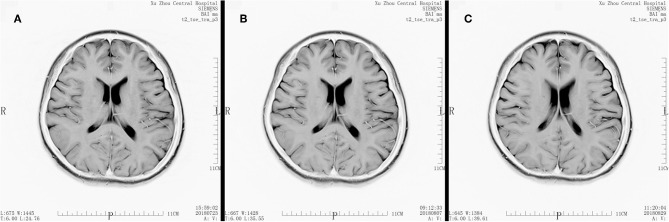
MRI T2 scan result of a patient. **(A)** Scan result before the surgery. **(B)** Scan result at 7 days after the surgery. **(C)** Scan result at 30 days after the surgery.

**Figure 3:**
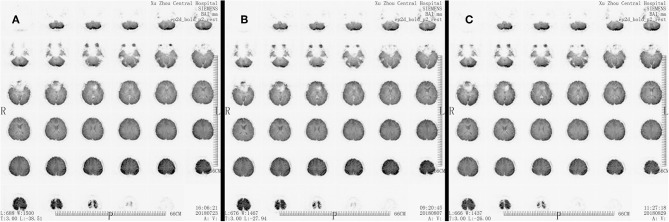
MRI Bold scan result of a patient. **(A)** Scan result before the surgery. **(B)** Scan result at 7 days after the surgery. **(C)** Scan result at 30 days after the surgery.

Medical image evaluation was performed by MRI (T1, T2, Bold) in all patients before the enrollment, and the results have been regarded as baseline. Any ischemic area occurrences were excluded from our study. Another two examination time points were 7 days after surgery and 1 month after surgery (Figures 1–3).

### Anesthesia

All patients received intramuscular injection of morphine 0.1 mg/kg and scopolamine 0.3 mg 30 min before surgery. After entering the operating room and opening the peripheral venous access, the mean arterial pressure was monitored by a radial arterial catheter under local anesthesia. Intravenous intubation was induced by anesthesia with midazolam 0.05 mg/kg, propofol 1 mg/kg, sufentanil 5 μg/kg, and cis-atracurium 0.3 mg/kg. Respiratory rate 12 times/min, tidal volume 8–10 ml/kg, inhaled oxygen concentration 80%, flow rate 1 L/min, and end-expiratory carbon dioxide partial pressure at 35–45 mmHg were maintained. A double-chamber central venous catheter was placed through the right internal jugular vein for central venous pressure monitoring. Continuous infusion of propofol 6–8 mg·kg^−1^·h^−1^ and inhalation of 1–1.5 MAC sevoflurane were done to maintain anesthesia, and sufentanil 0.5–1 μg/kg was added before incision of skin and extracorporeal circulation. The depth of anesthesia was maintained at the bispectral value of EEG 40–60.

Heparin was administered 3 mg/kg before extracorporeal circulation, allowing activated prothrombin time to exceed 480 s. The dose of fish sperm antagonism at the end of the extracorporeal circulation was 1 to 1.5:1 of the heparin dose, so that the prothrombin time value reached or approached the preoperative baseline value. During the operation, the volume was adjusted and/or vasoactive drugs (norepinephrine, dopamine, milrinone, nitroglycerin, etc.) were used according to hemodynamic parameters and transesophageal echocardiography to maintain an average arterial pressure of not <60 mmHg. After the operation, the patient was admitted to the intensive care unit (ICU) with tracheal catheter and was given analgesia after intravenous operation (the analgesic solution was sufentanil 2 μg/kg + tropisetron 10 mg, diluted to 100 ml with 0.9% sodium chloride solution). The continuous infusion dose was 0.04 μg·kg^−1^·h^−1^, the single additional dose was 0.02 μg/kg, and the locking time was 15 min.

### Measurements and Laboratory Data

To measure S100β, interleukin (IL)-6, matrix metalloproteinase (MMP)-9, and tumor necrosis factor (TNF)-α, venous blood samples were obtained at three time points: before anesthesia (T0), immediately after surgery (T1), and immediately after extubation (T2). Five milliliters of venous blood from the patient was taken and centrifuged, and the supernatant was separated and stored at −80°C in the refrigerator. Inflammatory factors were detected by enzyme-linked immunosorbent assay (Abclonal Biotechnology Co., Ltd., USA).

### Evaluation of Cognitive Function

Neuropsychiatric tests were performed by a special person 1 day before surgery and 7 days after surgery. Patients were screened with MMSE, and MMSE ≤23 points were excluded. To evaluate the possible learning effects in the test, we recruited volunteers with similar age levels to evaluate cognitive function at the same time using the Montreal Cognitive Assessment (MoCA) scale. The inclusion and exclusion criteria for subjects in the normal control group were the same as those for the patients, but no anesthesia and surgery were performed. POCD was diagnosed with Z-Score comprehensive scoring method recommended by the International Study of Postoperative Cognitive Dysfunction Group (ISPOCD group) (9). Specifically, (1) the differences between the two groups before and after the neuropsychological scale test were calculated, and the average value (M) was taken as the learning effect, wherein the learning effect was defined as the repeated estimation of the same scale test that may result in an increase in the false score of the patient due to familiarity with the scale content. (2) The differences between the two scales of the subject were also calculated, and the learning effect M was subtracted from the actual difference. Z = the actual difference of the score of a certain scale of the subject/the standard deviation of the difference of the score of the normal control of the same kind of scale. The larger the score of Z, the greater the score of the test after the test was lower than the preoperative drop. Z ≥ 1.96 was judged as abnormal. If there were not less than two items in the test abnormality ≥1.96, then POCD would be diagnosed. The learning effect of the normal control group is shown in Table 1.

**Table 1:** Learning effect of MoCA test score in normal control group.

**Day 1**	**Day 7**	**Leaning effect**
26.60 ± 1.28	27.73 ± 1.05	1.13 ± 0.68

### Medical Imaging Evaluation

#### Statistical Analysis

Statistical analysis was performed using SPSS l9.0. All measures were tested for normality and homogeneity of variance, expressed as mean ± standard deviation ($\bar{\;\chi \;}$±s), and the *t*-test or analysis of variance was used for comparison between groups, and the multiple comparison between groups was performed by SNK method (or LSD method); the count data were described by percentage, and the comparison between groups was performed by χ^2^ test, which were two-sided tests. *P* < 0.05 was considered statistically significant.

## Results

### Patient Characteristics

**Figure 4:**
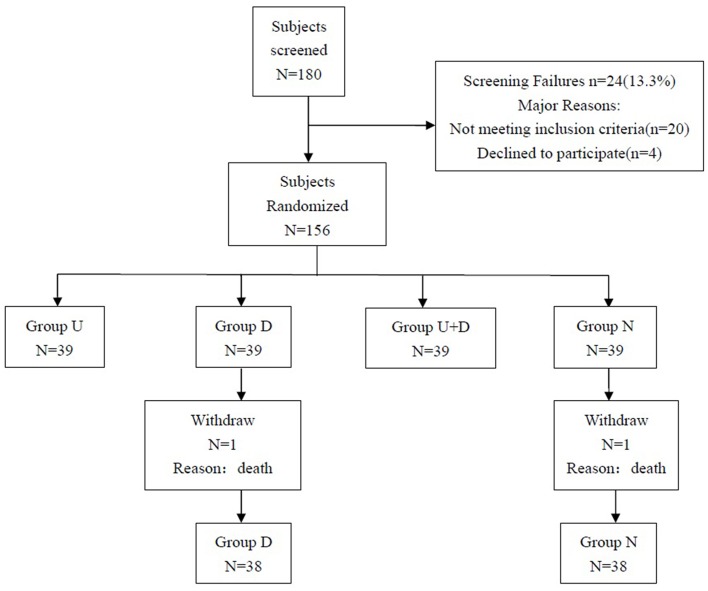
Patient recruitment flowchart.

The study plan included 180 patients, and a total of 156 patients were eventually enrolled, including 39 patients in group U, 39 patients in group D, 39 patients in group U+D, and 39 patients in group N. One patient died in group D and group N, and a total of 154 patients completed the study (Figure 4).

The MoCA score on the first day of group N was 26.60 ± 1.28, the MoCA score on the 7th day was 27.73 ± 1.05, and the learning effect was 1.13 ± 0.68.

The basic characteristics of the patients before surgery, such as gender, age, BMI, education level, MMSE score, and duration of surgery and tube time, were not statistically significant (*P* > 0.05, Table 2).

**Table 2:** Basic characteristics of patients.

	**Group U**	**Group D**	**Group U+D**	**Group N**	***P***
Gender (male/female)	17/22	17/19	16/22	18/20	0.957
Age (year)	70.6 ± 4.4	69.8 ± 5.1	69.6 ± 5.0	70.0 ± 4.9	0.819
BMI (kg/m^2^)	24.5 ± 3.1	22.8 ± 3.3	23.2 ± 4.2	24.9 ± 4.8	0.931
Education (year)	8.8 ± 3.4	10.0 ± 2.8	11.9 ± 3.5	9.1+3.6	0.737
MMSE scores	27.1 ± 3.6	26.8 ± 3.2	26.2 ± 3.0	26.5 ± 3.2	0.993
Length of surgery (min)	211.8 ± 23	235.2 ± 31	202.7 ± 22	224.3 ± 25	0.546
Length of CPB (min)	101.6 ± 9	120.8 ± 11	98.1 ± 7	115.2 ± 10	0.104
Endotracheal tube time (h)	24.5 ± 3.2	23.8 ± 3.4	25.1 ± 4.1	25.5 ± 4.6	0.971

*There was no significant difference in the basic characteristics (Gender, Age, BMI, Education), MMSE score, length of operation and time of catheterization among the groups before operation (p > 0.05)*.

The MoCA scores were significantly lower in the four groups on the seventh day after surgery compared with the day before surgery: group U 25.46 ± 1.99 vs. 26.58 ± 1.39, group D 25.34 ± 1.93 vs. 26.73 ± 1.48, group U+D 25.61 ± 2.17 vs. 26.74 ± 1.41, group N 24.68 ± 1.72 vs. 26.71 ± 1.52; the differences were statistically significant (*P* < 0.05). Compared with group N, the incidence of POCD decreased significantly in group U, group D, and group U+D with statistical significance (*P* < 0.05), but there was no significance between group U, group D, and group U+D (*P* > 0.05, Table 3).

**Table 3:** Comparison of MoCA score and POCD incidence in four groups.

	**1 day preoperation**	**7th day postoperation**	**Case of POCD**	**Incidence (%)**
Group U	26.58 ± 1.39	25.46 ± 1.99^c^	7 (*n* = 39)	17.9^a^
Group D	26.73 ± 1.48	25.34 ± 1.93^c^	6 (*n* = 38)	15.8^a^
Group U+D	26.74 ± 1.41	25.61 ± 2.17^c^	4 (*n* = 39)	10.3^a^
Group *N*	26.71 ± 1.52	24.68 ± 1.72^c^	12 (*n* = 38)	31.6

a *P < 0.05 vs. Group N*;

c *P < 0.05 vs. 1 day preoperation*.

*Compared with 1 day preoperation, the MoCA scores of the four groups decrease significantly on the 7th day postoperation (p < 0.05); Compared with group N, the incidence of POCD in group U, group D, and group U+D decreased significantly (p < 0.05). There was no significant difference among group U, group D, and group U+D (p > 0.05)*.

Compared with T0, S100β content was significantly increased at T1 and T2, and the difference was statistically significant (*P* < 0.05, Table 4).

**Table 4:** Concentration of Inflammatory factors of each group.

	**S100β** **(μg/L)**			**IL-6 (pg/mL)**			**TNF-α** **(pg/mL)**			**MMP-9 (ng/mL)**		
	**T0**	**T1**	**T2**	**T0**	**T1**	**T2**	**T0**	**T1**	**T2**	**T0**	**T1**	**T2**
Group U	0.11 ± 0.036	1.48 ± 0.14^a^^b^^c^	1.22 ± 0.12^a^^b^^c^	48.52 ± 7.22	239.54 ± 32.36^a^^b^^c^	133.79 ± 29.41^a^^b^^c^	22.32 ± 5.90	40.41 ± 9.31^a^^b^^c^	32.82 ± 7.62^abc^	254.23 ± 48.31	538.41 ± 58.45^a^^b^^c^	386.05 ± 50.17^a^^b^^c^
Group D	0.10 ± 0.03	1.58 ± 0.22^a^^b^^c^	1.18 ± 0.12^a^^b^^c^	47.69 ± 7.31	245.79 ± 31.95^a^^b^^c^	145.63 ± 28.13^a^^b^^c^	23.19 ± 5.75	42.34 ± 9.77^a^^b^^c^	34.45 ± 8.37^a^^b^^c^	260.61 ± 44.00	569.13 ± 53.63^a^^b^^c^	399.03 ± 52.11^a^^b^^c^
Group U+D	0.10 ± 0.03	1.26 ± 0.20^a^^b^	1.01 ± 0.15^a^^b^	48.74 ± 7.69	201.15 ± 27.35^a^^b^	92.33 ± 25.89^a^^b^	22.85 ± 4.96	34.89 ± 7.38^a^^b^	28.62 ± 6.52^a^^b^	258.38 ± 41.74	408.10 ± 48.07^a^^b^	314.36 ± 44.11^a^^b^
Group N	0.09 ± 0.03	2.14 ± 0.36^a^^c^	1.62 ± 0.23^a^^c^	48.01 ± 6.99	294.24 ± 39.61^a^^c^	202.39 ± 38.66^a^^c^	23.17 ± 5.64	58.94 ± 10.35^a^^c^	40.23 ± 8.85^a^^c^	248.82 ± 39.70	836.00 ± 61.11^a^^c^	561.13 ± 58.11^a^^c^

*Compared with T0*,

a *P < 0.05; compared with group N*,

b *P < 0.05;compared with U+D*,

c *P < 0.05*.

Compared with T0, IL-1 content was significantly increased at T1 and T2, and the difference was statistically significant (*P* < 0.05, Table 4);

Compared with T0, TNF-α content at T1 and T2 was significantly increased, and the difference was statistically significant (*P* < 0.05, Table 4).

Compared with T0, the MMP-9 content was significantly increased at T1 and T2, and the difference was statistically significant (*P* < 0.05, Table 4).

**Figure 5:**
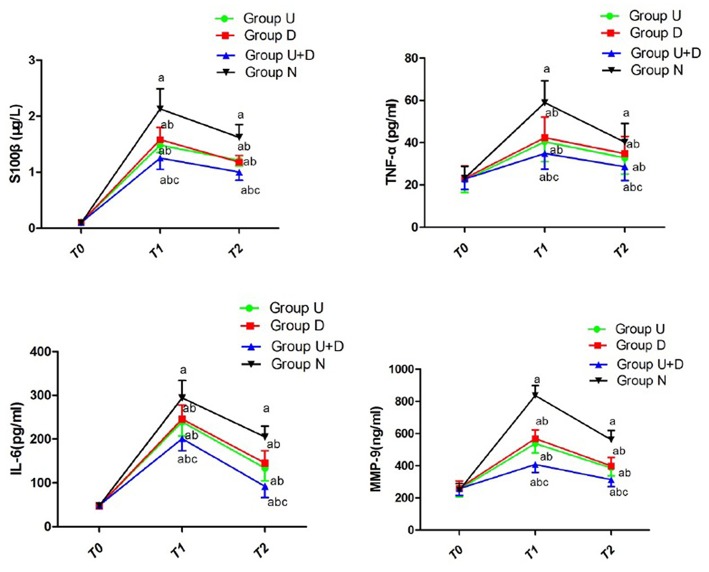
Concentration of S100β, TNF-α, IL-6, and MMP-9 of each group; compared with T0, ^a^*P* < 0.05; compared with group N, ^b^*P* < 0.05; compared with U+D, ^c^*P* < 0.05.

Compared with group N, the levels of S100β, IL-6, TNF-α, and MMP-9 in group U, group D, and group U+D were significantly decreased. Compared with U+D group, the contents of S100β, IL-6, TNF-α, and MMP-9 in U group, D group, and N group were significantly increased. The differences were statistically significant (*P* < 0.05, Table 4, Figure 5).

## Discussion

With the continuous development of surgical techniques, extracorporeal circulation techniques, and perioperative management, the mortality rate of cardiac surgery is decreasing year by year, but POCD remains a common problem in cardiac surgery. Heart valve replacement surgery is performed under cardiopulmonary bypass (CPB), and it is considered that CPB is one of the important factors causing POCD (11). The reason is that a large amount of inflammatory factors can be released after CPB, thus a large amount of inflammatory mediators are generated, causing a systemic inflammatory reaction. The inflammatory response is currently considered to be the most important mechanism for the occurrence of POCD. Clinically, anti-inflammatory drugs, such as ulinastatin and parecoxib, can reduce the level of inflammatory factors and reduce the incidence of POCD to some degree (8, 12). Animal studies have found that inhibition of key enzymes in inflammatory response, use of inflammatory factor antagonists, regulation of the peripheral immune system, and regulation of neurohumoral mechanisms can alleviate neuroinflammatory reactions and improve cognitive impairment (13–15).

In this study, the effect of ulinastatin combined with dexmedetomidine on POCD in patients undergoing cardiac surgery was evaluated. It was found that compared with the other three groups, the levels of inflammatory factors S100β, IL-6, MMP-9, and TNF- α were significantly decreased after the combination of the two drugs, and the incidence of POCD had a downward trend. This suggests that the combination of ulinastatin and dexmedetomidine is more conducive to the outcome of POCD in heart valve surgery patients.

Surgical trauma activates the immune system, leading to a perioperative inflammatory response. Increased levels of proinflammatory factors (such as IL-6 and TNF-α) in the brain increases blood-brain barrier permeability, inducing intracerebral inflammatory response or directly damaging neurons, causing autoimmune reactions in the brain, which may aggravate nerve collapse and neuronal damage (16). Sl00β protein is a kind of nerve tissue protein, which is extremely low in normal human serum. When nerve cells are damaged or blood-brain barrier permeability is increased, serum S100β protein can be detected to be abnormally increased. Farsak et al. (17) monitored the level of Sl00β protein during perioperative period and recorded neuropsychiatric scores before and after surgery, finding that Sl00β protein content has a good correlation with neuropsychiatric score. MMP-9 is a new target found in the research of the pathogenesis of nervous system diseases recently, which is mainly synthesized and secreted by astrocytes, microglia, epithelial cells, granulocytes, and neurons. MMP-9 is converted from an proenzyme form to an active state involved in the development of the disease. The study found that (18) in the blood of patients with central nervous system diseases (ischemic cerebrovascular disease, multiple sclerosis, Alzheimer's disease, malignant glioma, etc.), the activity of MMP-9 was significantly increased.

The protective effect of dexmedetomidine on the brain has been reported in recent years. Su et al. (19) found that the use of low-dose dexmedetomidine in elderly patients significantly reduces postoperative delirium possibly because dexmedetomidine inhibits the release of inflammatory factors. Ulinastatin can inhibit systemic inflammatory response and reduce neuronal apoptosis, thereby improving the body's ability to learn and remember, whose protective effect on major organs throughout the body during CPB surgery is widely recognized. In this study, a low-dose dexmedetomidine 0.4 μg/kg/h continuous pumping till 2 h before extubation combined with ulinastatin 20,000 units/kg pumping was selected, and the effect was more than that of any one drug alone, suggesting that the two drugs have a good synergistic effect in improving the POCD of heart valve surgery patients. Our study also recommended this two-drug application on perioperative period of cardiac surgeries to protect patients from POCD.

Of course, the study also has some shortcomings. First, the study lasted for a short period of time and was only followed up to 7 days after surgery; however, studies have shown that the incidence of POCD in heart patients is still 12–30% 1 month after surgery (20). Second, this study only used MMSE and MoCA for scale, which were simpler than ISPOCD and not accurate enough (21). Finally, the sample size of this study was small, observing the trend only, which was not enough to form a conclusion, still requiring further large-scale clinical research.

## Data Availability Statement

All datasets generated for this study are included in the article/supplementary material.

## Ethics Statement

The studies involving human participants were reviewed and approved by the Ethics Committee of Xuzhou Central Hospital. The patients/participants provided their written informed consent to participate in this study.

## Author Contributions

MZ and YL designed this study and wrote the manuscript. CW, TJ, YZ, and YL participated in the data acquisition and perioperative management. LW and JY designed this study and reviewed the manuscript.

### Conflict of Interest

The authors declare that the research was conducted in the absence of any commercial or financial relationships that could be construed as a potential conflict of interest.

## References

[1] Price CC Tanner JJ Schmalfuss I Garvan CW Gearen P Dickey D. A pilot study evaluating presurgery neuroanatomical biomarkers for postoperative cognitive decline after total knee arthroplasty in older adults. Anesthesiology. (2014) 120:601–13. 10.1097/ALN.0000000000000080 24534857 PMC3930070

[2] van Harten AE Scheeren TW Absalom AR. A review of postoperative cognitive dysfunction and neuroinflammation associated with cardiac surgery and anaesthesia. Anaesthesia. (2012) 67:280–93. 10.1111/j.1365-2044.2011.07008.x 22321085

[3] Mashour GA Woodrum DT Avidan MS. Neurological complications of surgery and anaesthesia. Br J Anaesth. (2015) 114:194–203. 10.1093/bja/aeu296 25204699

[4] Skvarc DR Berk M Byrne LK Dean OM Dodd S Lewis M. Post-operative cognitive dysfunction: an exploration of the inflammatory hypothesis and novel therapies. Neurosci Biobehav Rev. (2018) 84:116–33. 10.1016/j.neubiorev.2017.11.011 29180259

[5] Ma Y Cheng Q Wang E Li L Zhang X. Inhibiting tumor necrosis factor-alpha signaling attenuates postoperative cognitive dysfunction in aged rats. Mol Med Rep. (2015) 12:3095–100. 10.3892/mmr.2015.3744 25955232

[6] Sun L Dong R Xu X Yang X Peng M. Activation of cannabinoid receptor type 2 attenuates surgery-induced cognitive impairment in mice through anti-inflammatory activity. J Neuroinflammation. (2017) 14:138. 10.1186/s12974-017-0913-7 28724382 PMC5518095

[7] Atal SS Atal S Ulinastatin - a newer potential therapeutic option for multiple organ dysfunction syndrome. J Basic Clin Physiol Pharmacol. (2016) 2:91–9. 10.1515/jbcpp-2015-0003 26565549

[8] Lv ZT Huang JM Zhang JM Zhang JM Guo JF Chen AM Effect of ulinastatin in the treatment of postoperative cognitive dysfunction: review of current literature. BioMed Res Int. (2016) 2016:2571080 10.1155/2016/2571080 27597957 PMC5002304

[9] Cheng XQ Mei B Zuo YM Wu H Peng XH Zhao Q. A multicentre randomised controlled trial of the effect of intra-operative dexmedetomidine on cognitive decline after surgery. Anaesthesia. (2019) 74:741–50. 10.1111/anae.14606 30835822

[10] Xu G Li LL Sun ZT Zhang W Han XP. Effects of dexmedetomidine on postoperative cognitive dysfunction and serum levels of b-amyloid and neuronal microtubule-associated protein in orthotopic liver transplantation patients. Ann Transplant. (2016) 21:508–15. 10.12659/AOT.899340 27527391

[11] Gao L Taha R Gauvin D Othmen LB Wang Y Blaise G. Postoperative cognitive dysfunction after cardiac surgery. Chest. (2005) 128:3664–70. 10.1378/chest.128.5.3664 16304328

[12] Zhu YZ Yao R Zhang Z Xu H Wang LW. Parecoxib prevents early postoperative cognitive dysfunction in elderly patients undergoing total knee arthroplasty: a double-blind, randomized clinical consort study. Medicine. (2016) 95:e4082. 10.1097/MD.0000000000004082 27428192 PMC4956786

[13] Terrando N Yang T Wang X Fang J Cao M Andersson U. Systemic HMGB1 neutralization prevents postoperative neurocognitive dysfunction in aged rats. Front Immunol. (2016) 7:441. 10.3389/fimmu.2016.00441 27822212 PMC5075578

[14] Fan D Li J Zheng B Hua L Zuo Z. Enriched environment attenuates surgery-induced impairment of learning, memory, and neurogenesis possibly by preserving BDNF expression. Mol Neurobiol. (2016) 53:344–54. 10.1007/s12035-014-9013-1 25432890

[15] Chiang N Dalli J Colas RA Serhan CN. Identification of resolvin D2 receptor mediating resolution of infections and organ protection. J Exp Med. (2015) 212:1203–17. 10.1084/jem.20150225 26195725 PMC4516788

[16] Brown JA Codreanu SG Shi M Sherrod SD Markov DA Neely MD. Metabolic consequences of inflammatory disruption of the blood-brain barrier in an organ-on-chip model of the human neurovascular unit. J Neuroinflammation. (2016) 13:306. 10.1186/s12974-016-0760-y 27955696 PMC5153753

[17] Farsak B Gunaydin S Yorgancioglu C Zorlutuna Y Elevated levels of S100 beta correlate with neurocognitive outcome after cardiac surgery. J Cardiovasc Surg. (2003) 44:31–5. 12627069

[18] Bruno MA Mufson EJ Wuu J Cuello AC. Increased matrix metallopmteinase9 activity in mild cognitive impairment. J Neuropathol Exp Neurol. (2009) 68:1309–18. 10.1097/NEN.0b013e3181c22569 19915485 PMC2810197

[19] Su X Meng ZT Wu XH Cui F Li HL Wang DX. Dexmedetomidine for prevention of delirium in elderly patients after non-cardiac surgery: a randomised, double-blind, placebo-controlled trial. Lancet. (2016) 388:1893–902. 10.1016/S0140-6736(16)30580-3 27542303

[20] Hogue CW Jr Palin CA Arrowsmith JE. Cardiopulmonary bypass management and neurologic outcomes: an evidence—based appraisal of current practices. Anesth Analg. (2006) 103:21–37. 10.1213/01.ANE.0000220035.82989.79 16790619

[21] Moiler JT Cluitmans P Rasmussen LS Houx P Rasmussen H Canet J Long—term postoperative cognitive dysfunction in the elderly: ISPOCD l study. ISPOCD investigators. The International Study of Postoperative Cognitive Dysfunction. Lancet. (1998)35:857–61. 10.1016/S0140-6736(97)07382-0 9525362

